# Identification of two residues within the NS1 of H7N9 influenza A virus that critically affect the protein stability and function

**DOI:** 10.1186/s13567-018-0594-y

**Published:** 2018-10-01

**Authors:** Song Wang, Lanlan Zhang, Rong Zhang, Xiaojuan Chi, Zhou Yang, Yanhui Xie, Sicheng Shu, Yuan Liao, Ji-Long Chen

**Affiliations:** 1 Key Laboratory of Fujian-Taiwan Animal Pathogen Biology, College of Animal Sciences, Fujian Agriculture and Forestry University, Fuzhou, China; 2 CAS Key Laboratory of Pathogenic Microbiology and Immunology, Institute of Microbiology, Chinese Academy of Sciences, Beijing, China

## Abstract

The emerging avian-origin H7N9 influenza A virus, which causes mild to lethal human respiratory disease, continues to circulate in China, posing a great threat to public health. Influenza NS1 protein plays a key role in counteracting host innate immune responses, allowing the virus to efficiently replicate in the host. In this study, we compared NS1 amino acid sequences of H7N9 influenza A virus with those of other strains, and determined NS1 protein variability within the H7N9 virus and then evaluated the impact of amino acid substitutions on ability of the NS1 proteins to inhibit host innate immunity. Interestingly, the amino acid residue S212 was identified to have a profound effect on the primary function of NS1, since S212P substitution disabled H7N9 NS1 in suppressing the host RIG-I-dependent interferon response, as well as the ability to promote the virus replication. In addition, we identified another amino acid residue, I178, serving as a key site to keep NS1 protein high steady-state levels. When the isoleucine was replaced by valine at 178 site (I178V mutation), NS1 of H7N9 underwent rapid degradation through proteasome pathway. Furthermore, we observed that P212S and V178I mutation in NS1 of PR8 virus enhanced virulence and promoted the virus replication in vivo. Together, these results indicate that residues I178 and S212 within H7N9 NS1 protein are critical for stability and functioning of the NS1 protein respectively, and may contribute to the enhanced pathogenicity of H7N9 influenza virus.

**Electronic supplementary material:**

The online version of this article (10.1186/s13567-018-0594-y) contains supplementary material, which is available to authorized users.

## Introduction

Influenza A virus (IAV), whose genome is organized into eight single stranded negative-sense RNA segments that encode for at least 18 viral proteins [1], belongs to Orthomyxoviridae family and is subdivided into various subtypes according to surface glycoproteins, hemagglutinin (HA) and neuraminidase (NA). There are currently 18 known HA antigenic types and 11 known NA antigenic types with many possible combinations (subtypes) between HA and NA [2–4]. Since early 2013, a new emerging avian-origin H7N9 influenza A virus, whose zoonotic transmission has led to a total of 1567 laboratory-confirmed human infections as of 2 March 2018, has posed a considerable challenge to public health in China and raised great concerns in the world as this virus can cause severe infections in humans [5].

To date, the novel H7N9 influenza virus has been infecting humans with a high mortality rate of about 40%, indicating that H7N9 influenza viruses are much more pathogenic than 2009 pandemic H1N1 influenza virus and the seasonal H3N2 influenza virus [6, 7]. It has been suggested that several amino acid substitutions in PB2, PA, NP, HA, NA and NS1 may be responsible for promoting replication and pathogenicity of this virus in humans [8–14]. Although no sustained human-to-human transmission of H7N9 viruses has been observed, the genomic sequence changes in H7N9 virus, which may alter its virulence and augment the possibility of efficient transmission between humans, are also still of considerable concern. Importantly, it has been recently reported that some highly pathogenic H7N9 mutants were detected in birds, suggesting an increased threat to humans [14–16].

NS1 of IAV, a multifunctional nonstructural protein that is encoded by the shortest vRNA segment, is composed of the N-terminal RNA-binding domain and the C-terminal effector domain [17]. The NS1 protein is expressed early in viral replication cycle, and plays a key role in counteracting host innate immune responses through multiple mechanisms, including sequestering of double-stranded RNA, decreasing retinoic acid-inducible gene 1 (RIG-I) activation through interacting with RIG-I [18–20] or inhibiting tripartite motif family 25 (TRIM25)-mediated RIG-I ubiquitination [21], restraining the transcription and translation of host antiviral response genes, or directly modulating protein kinase R and 2′-5′-oligoadenylate synthetase (OAS) activity [17, 22].

The IAV NS1 protein is considered a major virulence factor, and an increasing number of studies have identified specific NS1 residues that are associated with virulence. For example, amino acid substitution G45R on the NS1 protein of A/Puerto Rico/8/1934 (H1N1) enhanced viral replication and increased virulence by inducing an earlier and robust proinflammatory cytokine response, while T49E mutation impairs its binding to TRIM25 and complex formation with RIG-I, thereby switching off its interferon antagonistic activity [23–25]. I64T, D189N and V194I mutations in NS1 protein of circulating H3N2 human influenza virus weakened NS1-mediated general inhibition of host protein synthesis by decreasing its interaction with cleavage and polyadenylation specificity factor 30 (CPSF30), leading to attenuated virulence and increased innate immune responses after the viral infection [26, 27]. In addition, the amino acid S42 of H5N1 influenza virus NS1 protein has an effect on resisting the host cell antiviral immune response via preventing the activation of the NF-κB and the IRF-3 pathways both mediated by the double-stranded RNA, and a single S42P mutation in NS1 protein could completely attenuate the virulence of A/Duck/Guangxi/27/03 (H5N1) virus in mice [28]. It has also been found that the human A/Hong Kong/156/1997 (H5N1) influenza virus NS1 F103L and M106I mutations could both increase IFN antagonism and virulence via increasing cytoplasmic NS1 expression as well as increased binding with host factors such as RIG-I [29]. Moreover, a distinct E172K substitution in the H7N9 NS1 protein could enhance virus replication in mammalian cells and mice through affecting SF2-ESE interaction that regulates splicing of NEP/NS1 mRNA [13].

In addition, previous studies have also shown that the amino acid substitution in the NS1 protein of IAV had effect on its post-translational modification and thus affected its biological functions and the virus replication. For example, influenza virus NS1 can be post-translationally modified by small ubiquitin-like modifier 1 (SUMO1), and two C-terminal residues K219 and K221 have been identified as SUMO1 acceptor sites of H5N1 NS1 protein. When the two lysines were replaced by arginines, NS1 protein lost its stability, which reduced its ability to suppress host protein expression and as a consequence, caused a retardation of virus growth [30]. Together, these data suggest that a single amino acid substitution in the IAV NS1 protein could alter its function and virulence. However, although it is well known that H7N9 IAV is a highly pathogenic virus for human, its evolution by point mutations and the precise mechanisms underlying its pathogenesis remains to be further determined.

In this study, we sought to identify the evolved residues that are important for H7N9 NS1 function. To this end, we aligned and compared NS1 amino acid sequences of influenza virus A/Anhui/1/2013 (H7N9) with those of four other influenza virus strains, A/Hong Kong/156/97 (H5N1), A/WSN/1933 (H1N1) (WSN), A/Puerto Rico/8/1934 (H1N1) (PR8) and A/California/04/2009 (H1N1) (CA04). Among them, H7N9 and H5N1 were highly pathogenic to human, while WSN, PR8 and CA04 were low pathogenic. Our experiments demonstrated that two crucial amino acids in H7N9 NS1 protein, I178 and S212, had profound effect on NS1 biological function. S212P mutation significantly impaired the antagonistic action of NS1 toward host innate immune response, and I178V mutation greatly decreased the stability of NS1 protein, resulting in NS1 degradation through proteasome pathway.

## Materials and methods

### Ethics statement

The animal protocol used in this study was approved by “the Regulation of College of Animal Sciences, Fujian Agriculture and Forestry University of Research Ethics Committee” (Permit Number PZCASFAFU2015003). All mouse experiments were carried out according to the Regulations for the Administration of Affairs Concerning Experimental Animals approved by the State Council of People’s Republic of China.

### Viruses and reagents

Influenza virus strains A/WSN/33 (H1N1), A/Puerto Rico/8/1934 (H1N1) and A/Puerto Rico/8/1934 deltaNS1 (PR8 delNS1) were propagated in specific-pathogen-free (SPF) chicken embryo as previously described [31]. The following antibodies were used in this study: anti-IAV NS1 and anti-IkBα (Santa Cruz Biotechnology, Santa Cruz, CA, USA); anti-Flag (Sigma-Aldrich, St. Louis, MO, USA); anti-STAT1 and anti-phospho-STAT1 (Tyr701), anti-NF-κB p65 and anti-phospho-NF-κB p65 (Ser536) (Cell Signaling Technology, Boston, MA, USA); anti-β-actin (Abcam, Cambridge, UK). Anti-IAV HA and anti-IAV NP were obtained as described previously [32]. The proteasome inhibitor MG132 was obtained from Merck (Darmstadt, Germany). The protein synthesis inhibitor cycloheximide (CHX) and the lysosome inhibitors NH_4_Cl and chloroquine were purchased from Sigma-Aldrich.

### Site-directed mutagenesis and plasmid construction

cDNA encoding NS1 (wild type) of A/Anhui/1/2013 H7N9 was synthesized and subcloned into the pNL-CMV vector with a Flag tag in the COOH terminus. The H7N9 NS1 mutants, NS1 (R55E), NS1 (H63Q), NS1 (E70K), NS1 (P87S), NS1 (S114P), NS1 (A143T), NS1 (I178V) and NS1 (S212P) were generated by site-directed mutagenesis with the QuickChange XL system (Stratagene, La Jolla, CA, USA).

### Rescue of recombinant influenza A viruses

PR8-WT virus and PR8 NS1 mutated virus were generated by reverse genetics as described previously [33]. Briefly, 293T cells were cotransfected with 1 μg of each of the eight plasmids by using Lipofectamine 3000 reagent (Invitrogen, San Diego, CA, USA). The supernatant was collected after 48 h of transfection and subsequently inoculated into 9-day-old embryonated chicken eggs. After 48 h of incubation, allantoic fluid containing the virus was harvested and stored at −80 °C.

### Cell culture and virus infection

293T, A549 and MDCK cells were purchased from American Type Culture Collection (Manassas, VA, USA). The cells were cultured in Dulbecco’s modified Eagle’s medium (DMEM) containing 10% fetal bovine serum (FBS) supplemented with penicillin (100 U/mL) and streptomycin (100 μg/mL). For virus infection, cells were infected with influenza virus at the indicated multiplicity of infection (MOI). After adsorption for 1 h at 37 °C, the cells were washed with phosphate-buffered saline (PBS) and cultured in DMEM containing 2 μg/mL of trypsin (Sigma-Aldrich).

### Mouse experiments

Female BALB/c mice (5–6 weeks old) were obtained from Shanghai SLAC Laboratory Animal Co., Ltd. (Shanghai, China). For virus infection, mice were anaesthetized and inoculated intranasally with 5 × 10^4^ PFU of PR8-WT virus or PR8 NS1 deleted virus. Mice were monitored for signs of disease and their body weight was measured every day for total 14 days or until the loss of 25% of their body weight, at which point they were euthanized. Survival of mice was monitored until 14 days post-infection and the survival rate for each virus was determined. For histopathological analysis and viral load determination in lungs, mice were euthanized and the lungs were removed for further analysis by hematoxylin and eosin (H&E) staining and plaque assay. Influenza virus infection of mice was carried out under enhanced BSL-2 (BSL-2+) conditions.

### RNA preparation, RT-PCR, and quantitative real-time PCR

Total RNA was extracted from cells using TRIzol reagent (Invitrogen). cDNA was synthesized using 2 μg of total RNA and Moloney murine leukemia virus (MMLV) reverse transcriptase (Promega, Madison, WI, USA), followed by PCR using rTaq DNA polymerase and quantitative PCR using SYBR Premix Ex Taq II (TaKaRa, Tokyo, Japan). The sequences of the primers used are available upon request.

### Cell extracts and Western blotting analysis

Cell lysates were prepared, and Western blotting was performed as previously described [31, 34]. Briefly, samples were separated on SDS-polyacrylamide gel, transferred onto a nitrocellulose membrane, and probed with antibodies as indicated.

### Plaque assay

Plaque assay was performed as previously described [35, 36]. Briefly, MDCK cells were infected with serial dilutions of the viruses. After an incubation period, cells were washed with PBS and overlaid with DMEM containing 1.5% low melting point agarose (Promega, Madison, WI, USA) and 2 μg/mL TPCK (tolylsulfonyl phenylalanyl chloromethyl ketone)-treated trypsin. After 72 h of incubation at 37 °C, number of plaques was counted.

### Histopathological analysis

Histopathological analysis was performed as described previously [31, 32]. In brief, mouse tissues were fixed in 4% paraformaldehyde and embedded in paraffin. Then, 5 μm thick sections were prepared and stained with H&E. Sections were examined under a Nikon Eclipse Ti-E microscope (Kobe, Japan).

### Statistical analysis

Data represent the mean values ± standard errors of the mean (s.e.m.). Statistical analysis was performed by Student’s *t* test. A level of *P* < 0.05 was considered statistically significant.

## Results

### Forced expression of H7N9 NS1 protein suppresses host innate immune response and promotes the viral replication

**Figure 1:**
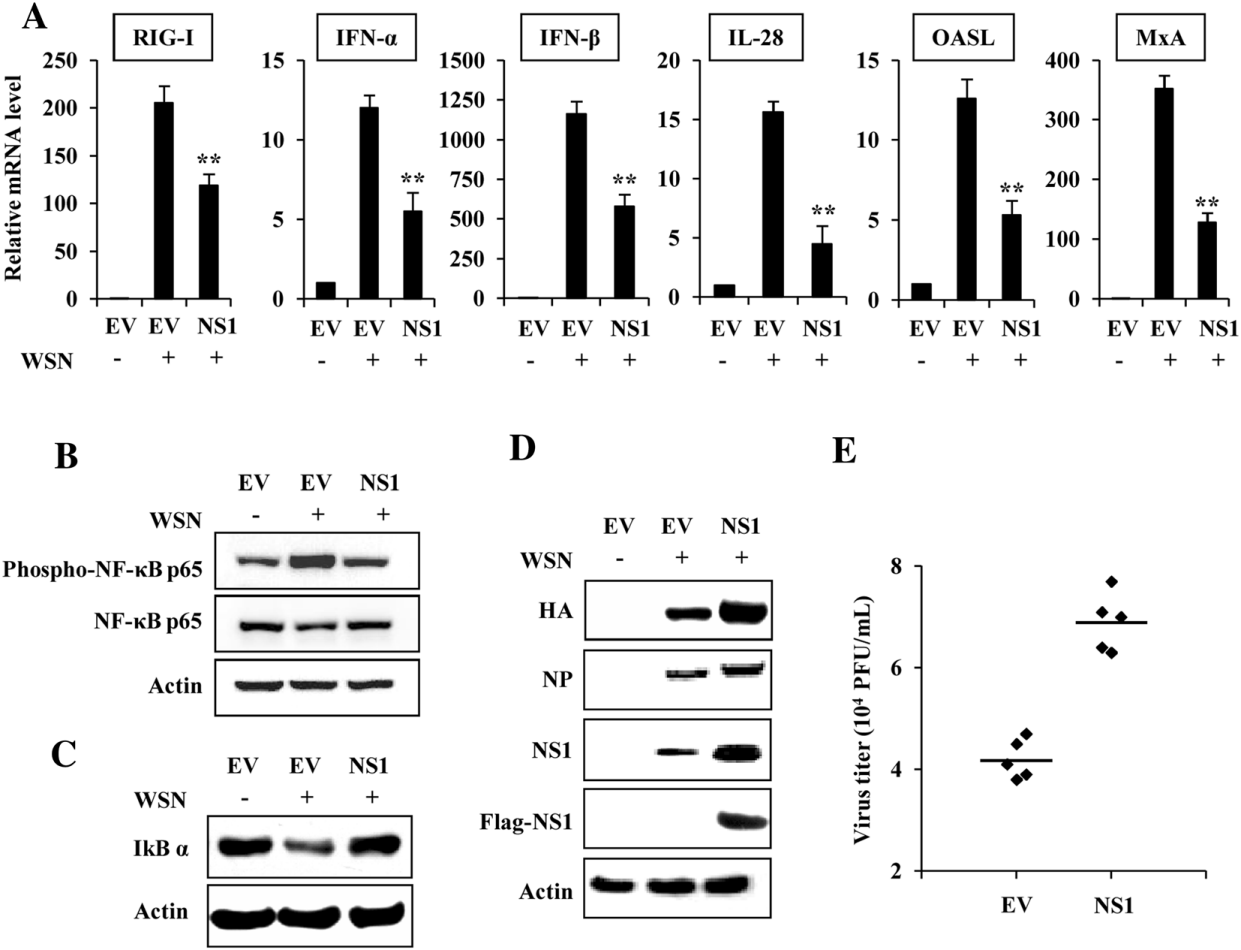
**H7N9 NS1 protein impairs host innate immune response. A** 293T cells transfected with plasmids expressing H7N9 NS1 or empty vector (EV) were infected with WSN virus (MOI = 1) for 12 h, followed by quantitative real-time PCR to detect the mRNA levels of the indicated genes. In each experiment, the value observed in mock-infected cells was normalized to 1. Plotted are the average levels from three independent experiments. The error bars represent the s.e.m. **P < 0.01. **B**–**D** 293T cells transfected with plasmids expressing H7N9 NS1 or EV were infected with WSN virus as described in (**A**). Then the cells were harvested and cell extracts were prepared for Western blotting using indicated antibodies. Shown are representative data of three independent experiments with similar results. **E** 293T cells transfected with plasmids expressing H7N9 NS1 or EV were treated as described in (**A**), and viral titers in the supernatants of the cells were examined by plaque assay.

Since H7N9 virus is highly pathogenic for human and IAV NS1 plays a key role in suppressing the host innate immunity, we analyzed the effect of forced expression of H7N9 NS1 on IAV-induced innate immune response and viral replication. 293T cells were transfected with plasmids expressing H7N9 NS1 or empty vector (EV) for 24 h, followed by WSN virus infection. As expected, the IAV infection induced the expressions of RIG-I, IFN-α, IFN-β, IL-28, OASL and MxA (Figure 1A). However, levels of these innate immune molecules were clearly reduced in cells expressing H7N9 NS1 as compared to those in EV-transfected cells, indicating that forced expression of H7N9 NS1 protein inhibits host innate immune response (Figure 1A). A previous study revealed that expression of NS1 protein can prevent virus-mediated activation of the NF-κB pathway [37], thus NF-κB activation was also examined. Results showed that NF-κB p65 was significantly phosphorylated after infection with WSN, but the virus-induced phosphorylation of NF-κB was reduced by forced expression of H7N9 NS1 protein (Figure 1B). In addition, the protein level of IκBα, the inhibitor of NF-κB, was reduced in the virus-infected cells (Figure 1C, lane 2), but overexpression of H7N9 NS1 restored IκBα level to a value close to that observed in non-infected cells (Figure 1C, lane 3), suggesting that NF-κB activation was blocked by the NS1.

We speculated that impaired host innate immunity would favor influenza virus replication. Indeed, our results showed that in H7N9 NS1 expressing cells infected with WSN, levels of the viral HA and NP proteins were much higher than those in control cells (Figure 1D). Similarly, we found that expression of H7N9 NS1 slightly enhanced the viral replication, as evidenced by increased virus titer in the supernatant of H7N9 NS1 expressing cells (Figure 1E). These data suggested that the H7N9 NS1 functions as a potent inhibitor antagonizing host innate immune response.

### S212P substitution significantly impairs antagonistic effect of H7N9 NS1 protein on host innate immune response

**Figure 2:**
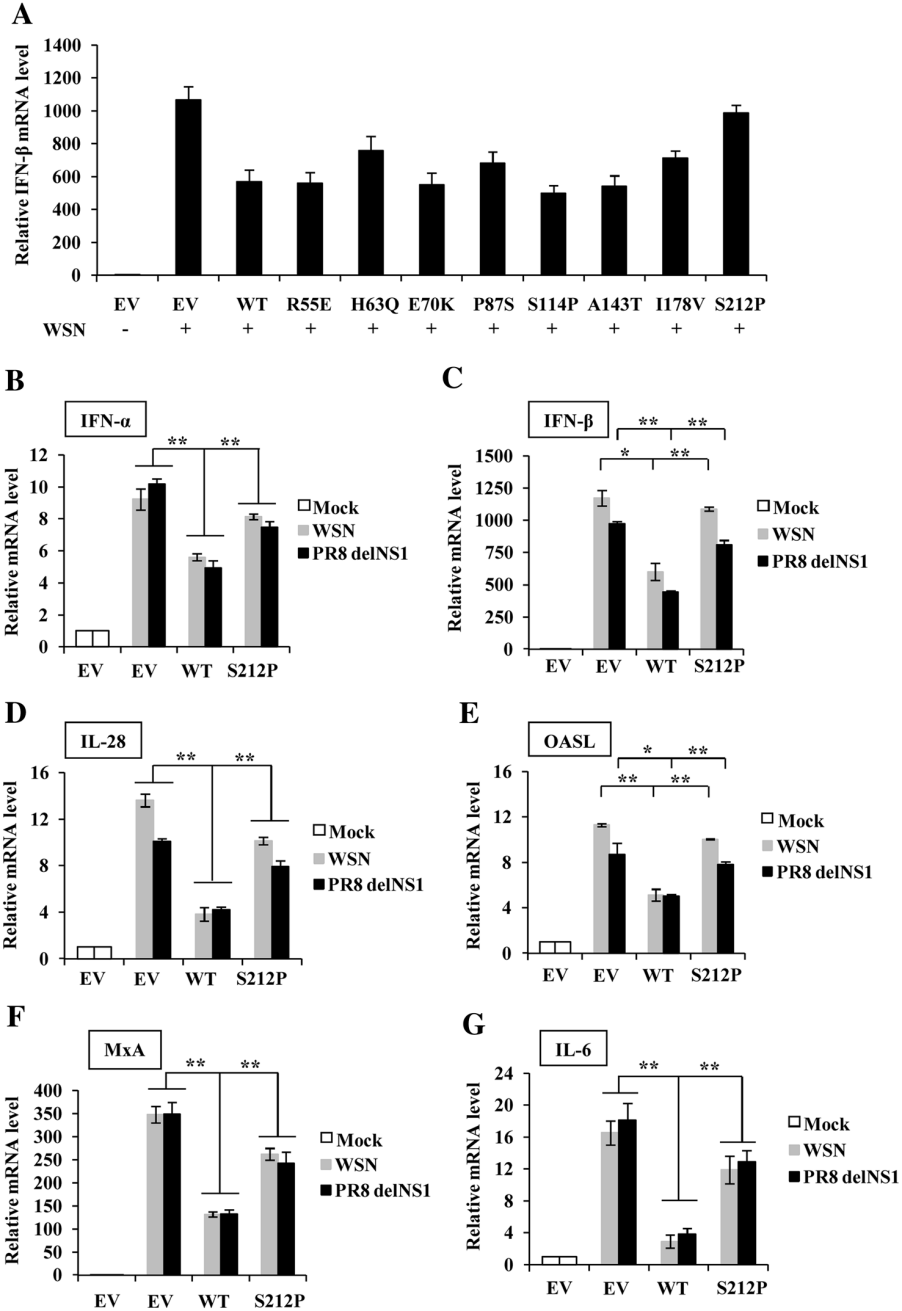
**H7N9 NS1-S212P mutation reduces its capacity to inhibit WSN and PR8 delNS1 virus-induced host antiviral response. A** 293T cells were transfected with plasmids expressing H7N9 NS1-WT (WT), NS1 mutants (R55E, H63Q, E70K, P87S, S114P, A143T, I178V, S212P) or EV for 24 h, then the cells were infected with WSN virus (MOI = 1) for 12 h, followed by quantitative real-time PCR to detect IFN-β mRNA expression levels. **B**–**G** After 293T cells were transfected with plasmids expressing H7N9 NS1-WT (WT), NS1-S212P (S212P) or EV for 24 h, the cells were then infected with WSN or PR8 delNS1 virus (MOI = 1) for 12 h, followed by quantitative real-time PCR to detect IFN-α (**B**), IFN-β (**C**), IL-28 (**D**), OASL (**E**), MxA (**F**) and IL-6 (**G**) mRNA levels. In each experiment, the value observed in mock-infected cells was normalized to 1. Plotted are the average levels from three independent experiments. The error bars represent the s.e.m. ^*^P < 0.05, ^**^P < 0.01.

To further assess variability in the H7N9 NS1 protein and identify new residues important for H7N9 NS1 function that may affect the pathogenicity of the virus, the amino acid sequences of H7N9 NS1 were aligned with those of four other influenza virus strains, A/Hong Kong/156/97 (H5N1), A/WSN/1933 (H1N1) (WSN), A/Puerto Rico/8/1934 (H1N1) (PR8) and A/California/04/2009 (H1N1) (CA04). H7N9 and H5N1 viruses were avian-origin and highly pathogenic to human, while WSN, PR8 and CA04 viruses were low pathogenic. We compared amino acids of NS1 between highly pathogenic and low pathogenic influenza viruses to determine single amino acid substitutions in the protein that could alter NS1 function and virulence of the virus. The complete alignment of these five NS1 protein sequences showed that there exist several substitutions which could distinguish the two avian-origin strains (H7N9 and H5N1) and the three human strains (WSN, PR8 and CA04). The substitutions were the following (avian_residue/Position/human_residue): R55E, H63Q, E70K, P87S, S114P, A143T, I178V, S212P (Additional file 1). To verify whether these amino acid changes contributed to the high pathogenicity of H7N9 virus to human, site-directed mutagenesis was carried out to generate the eight H7N9 NS1 mutants, R55E, H63Q, E70K, P87S, S114P, A143T, I178V and S212P, and wild type (WT) NS1 and NS1 mutants were transfected into 293T cells, and the cells were then infected with WSN virus to examine the effect of NS1 mutation on its antagonism against host innate immunity during the IAV infection. Interestingly, compared with the NS1 WT, NS1 S212P mutation caused the most significant increase in the IFN-β expression (Figure 2A), suggesting that the S212P mutation weakened the inhibition of host antiviral response by H7N9 NS1.

In addition to IFN-β, we observed that the expression of IFN-α, IL-28, MxA and OASL was also increased in NS1-S212P transfected cells comparing with that in NS1-WT transfected 293T cells after infection with WSN virus, while the expression of IL-29 and ISG15 was not influenced in these cells (Figures 2B–F, Additional file 2A). Similar results were obtained from experiments using A549 cell infected with WSN virus (Additional file 2B) or using 293T cell infected with PR8 virus (Additional file 2C). To exclude the effect of WSN or PR8 virus NS1, PR8 delNS1 virus was further used to repeat these experiments. Similarly, data showed that S212P mutation in H7N9 NS1 impaired its inhibitory effect on the expression of above antiviral factors (Figures 2B–F, Additional file 2D). Moreover, levels of inflammatory factors, including IL-6 and IL-1β, were also markedly affected by expression of NS1 S212P mutation (Figure 2G, Additional files 2E and F). Taken together, these results indicated that S212 residue in H7N9 NS1 protein could enhance its inhibitory effect on the expression of antiviral IFNs, ISGs and inflammatory factors.

### S212P mutation in H7N9 NS1 protein reduces its ability to suppress IAV-induced RIG-I expression and STAT1 activation

**Figure 3:**
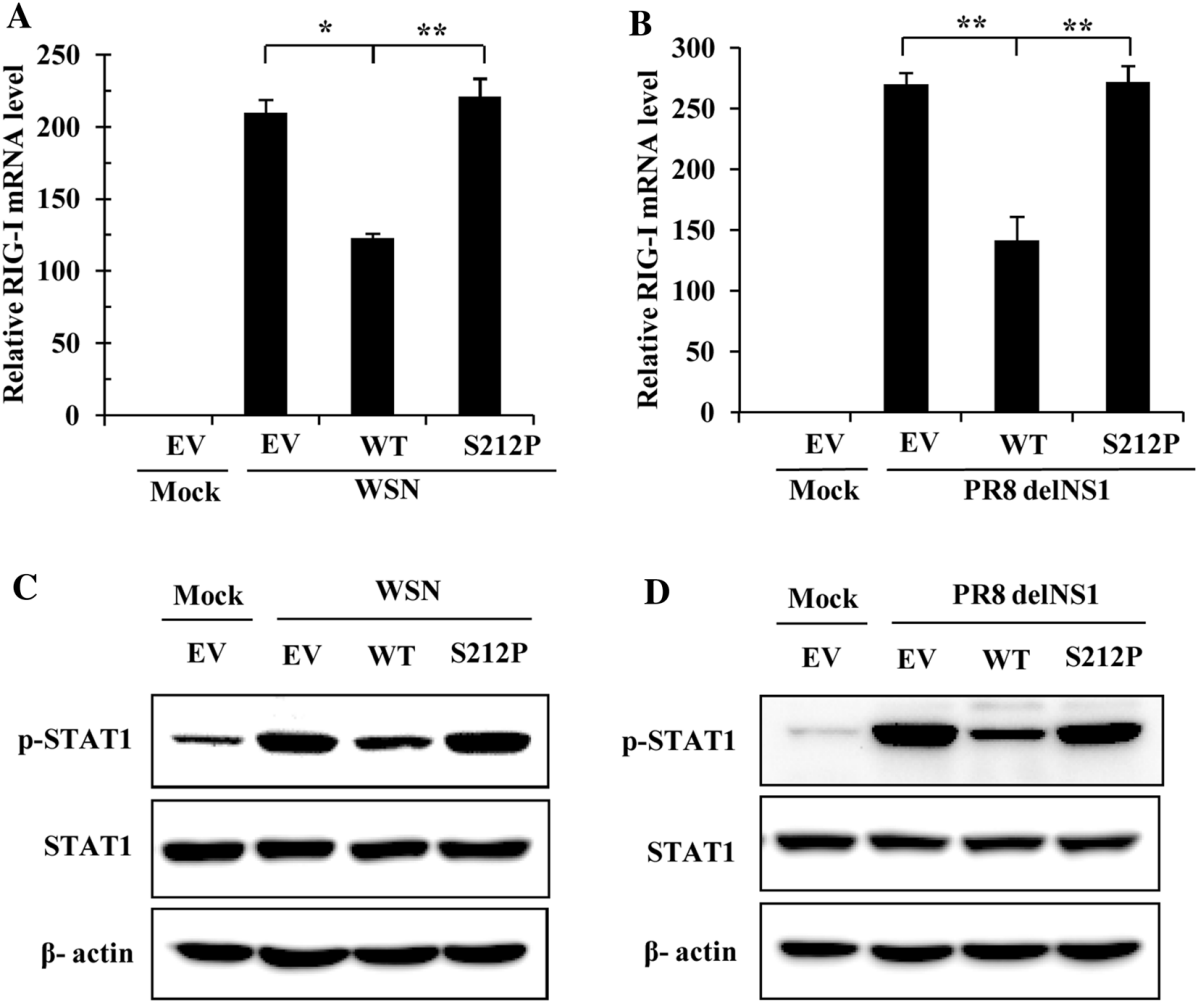
**H7N9 NS1 S212P mutation causes a decrease of its ability to suppress RIG-I expression and STAT1 activation. A**, **B** 293T cells transfected with plasmids expressing H7N9 NS1-WT (WT), NS1-S212P (S212P) or EV were infected with WSN (**A**) or PR8 delNS1 virus (**B**) (MOI = 1) for 12 h, followed by quantitative real-time PCR to detect RIG-I mRNA levels. In each experiment, the RIG-I level in mock-infected cells was normalized to 1. Plotted are the average levels from three independent experiments. The error bars represent the s.e.m. (**C**, **D**) 293T cells transfected with plasmids expressing H7N9 NS1-WT (WT), NS1-S212P (S212P) or EV were infected with WSN (**C**) or PR8 delNS1 virus (**D**) as described in (**A**, **B**), then cells were harvested and cell extracts were prepared for Western blotting using indicated antibodies. The results are representative of three independent experiments. ^*^P < 0.05, ^**^P < 0.01.

To further analyze whether S212P mutation in H7N9 NS1 protein affected its ability to counteract the innate immune responses, human 293T cells were transiently transfected with plasmids expressing WT NS1 or the NS1 mutant. Cells transfected with the EV were included as controls. At 24 h post-transfection (hpt), cells were mock infected or infected with WSN or PR8 delNS1 virus. Among the three most important pattern recognition receptors (PRRs) acting as sensors of influenza virus RNAs, only expression of RIG-I, but not MDA5 and TLR3, was influenced by the NS1-S212P mutant in cells infected with either WSN or PR8 delNS1 virus (Additional file 3). We observed that WT NS1 of H7N9 significantly inhibited IAV-induced RIG-I expression, but S212P amino acid substitution impaired its ability to inhibit the RIG-I expression (Figures 3A, B and Additional file 3). Since JAK-STAT signaling pathway plays a key role in host innate immune response and STAT1 activation enhances the transcription of numerous IFN stimulatory genes [38], we further examined the effect of H7N9 NS1 S212P mutation on STAT1 activation. After H7N9 NS1-WT was transfected into 293T cells, WSN or PR8 delNS1 induced STAT1 phosphorylation was inhibited to a great extent (Figures 3C and D). However, in NS1-S212P transfected cells, the phosphorylation of STAT1 was comparable to that in EV transfected cells (Figures 3C and D), which indicated that S212P mutation disables the NS1 in preventing STAT1 activation. Together, these experiments demonstrated that S212P mutation in H7N9 NS1 protein impaired its ability to suppress RIG-I/STAT1-mediated host innate immunity.

### S212P mutation of H7N9 NS1 protein results in a decrease in the viral replication

**Figure 4:**
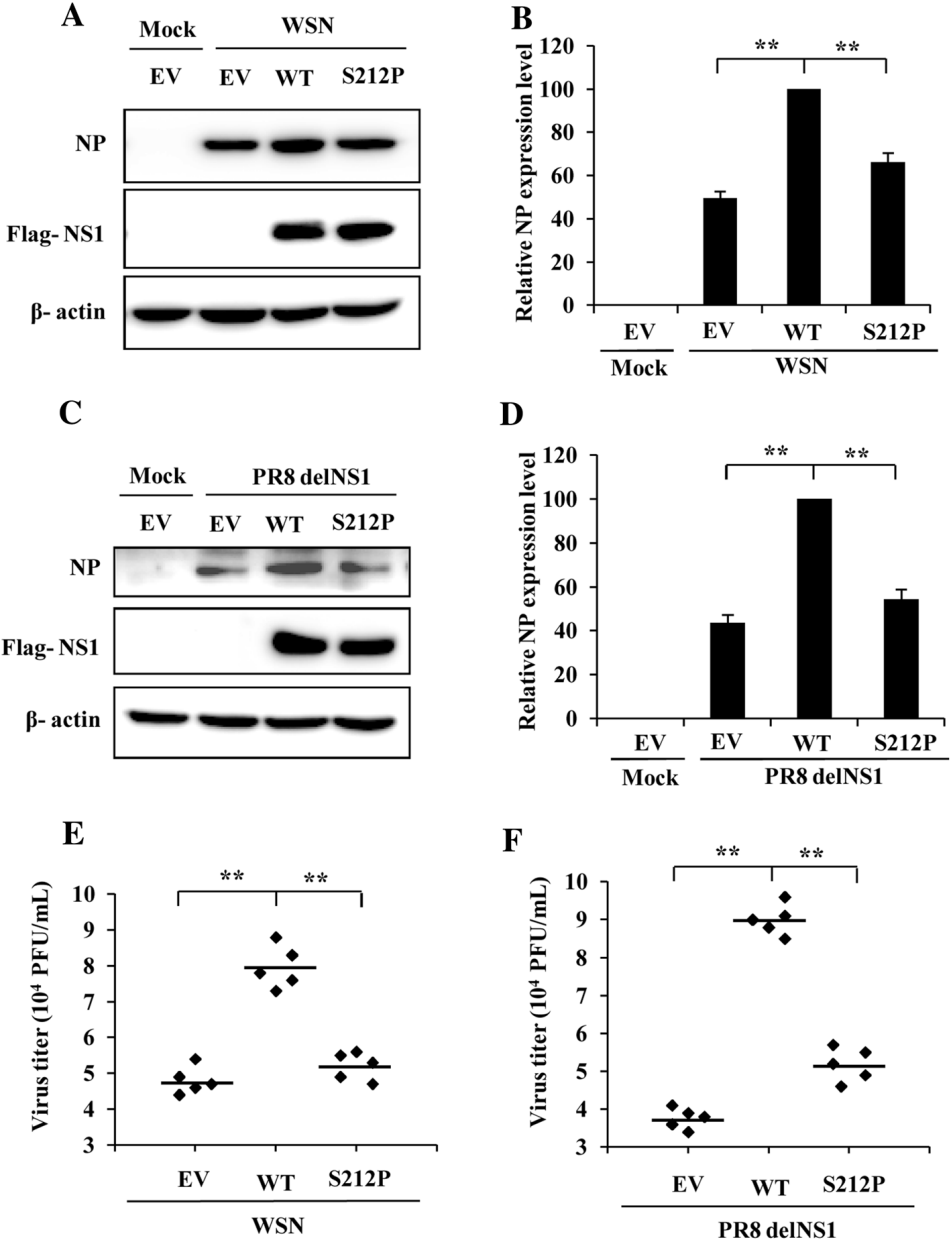
**S212P mutation of H7N9 NS1 results in a reduction in the viral replication. A** 293T cells transfected with plasmids expressing H7N9 NS1-WT (WT), NS1-S212P (S212P) or EV were infected with WSN (MOI = 1) for 12 h, followed by Western blotting to detect viral NP expression. **B** NP levels in (**A**) were quantitated by densitometry, and normalized to β-actin levels. In each experiment, the NP level in NS1-WT transfected cells is 100. **C** 293T cells transfected with plasmids expressing H7N9 NS1-WT (WT), NS1-S212P (S212P) or EV were infected with PR8 delNS1 virus (MOI = 1) for 12 h, followed by Western blotting to detect viral NP expression. **D** NP levels in (**C**) were quantitated by densitometry, and normalized to β-actin levels. In each experiment, the NP level in NS1-WT transfected cells is 100. **E**, **F** 293T cells transfected with plasmids expressing H7N9 NS1-WT (WT), NS1-S212P (S212P) or EV were infected with WSN (**E**) or PR8 delNS1 virus (**F**) (MOI = 1) for 12 h, and viral titers in the supernatants of the cells were examined by plaque assay. ^**^P < 0.01.

NS1 protein has been previously shown to be critical for the virulence of influenza virus [39]. Likewise, amino acid substitutions affecting the ability of NS1 to counteract the host innate immune response have been found to reduce the virulence of influenza virus, as well as its replication [26, 27]. To analyze whether S212P mutation affects virus replication, plasmids expressing NS1-WT or NS1-S212P were transiently transfected into 293T cells. Cells transfected with the EV were included as the control. At 24 hpt, cells were mock infected or infected with WSN or PR8 delNS1 virus for 12 h. Then the NP protein of influenza virus and virus titers in the cell supernatant were examined by Western blotting and plaque assay respectively. The results displayed that NP expression level of WSN virus or PR8 delNS1 virus was reduced to ~60% in cells expressing NS1-S212P mutant as compared to that in NS1-WT transfected cells (Figures 4A–D). Similarly, no matter infection with WSN virus or PR8 delNS1 virus, the virus titers in the supernatant derived from NS1-S212P transfected cells were decreased as compared with those derived from NS1-WT transfected cells (Figures 4E and F). These results suggest that S212P mutation of H7N9 NS1 causes a significant reduction in the NP protein expression and the virus replication.

### I178V mutation decreases the stability of H7N9 NS1 protein

**Figure 5:**
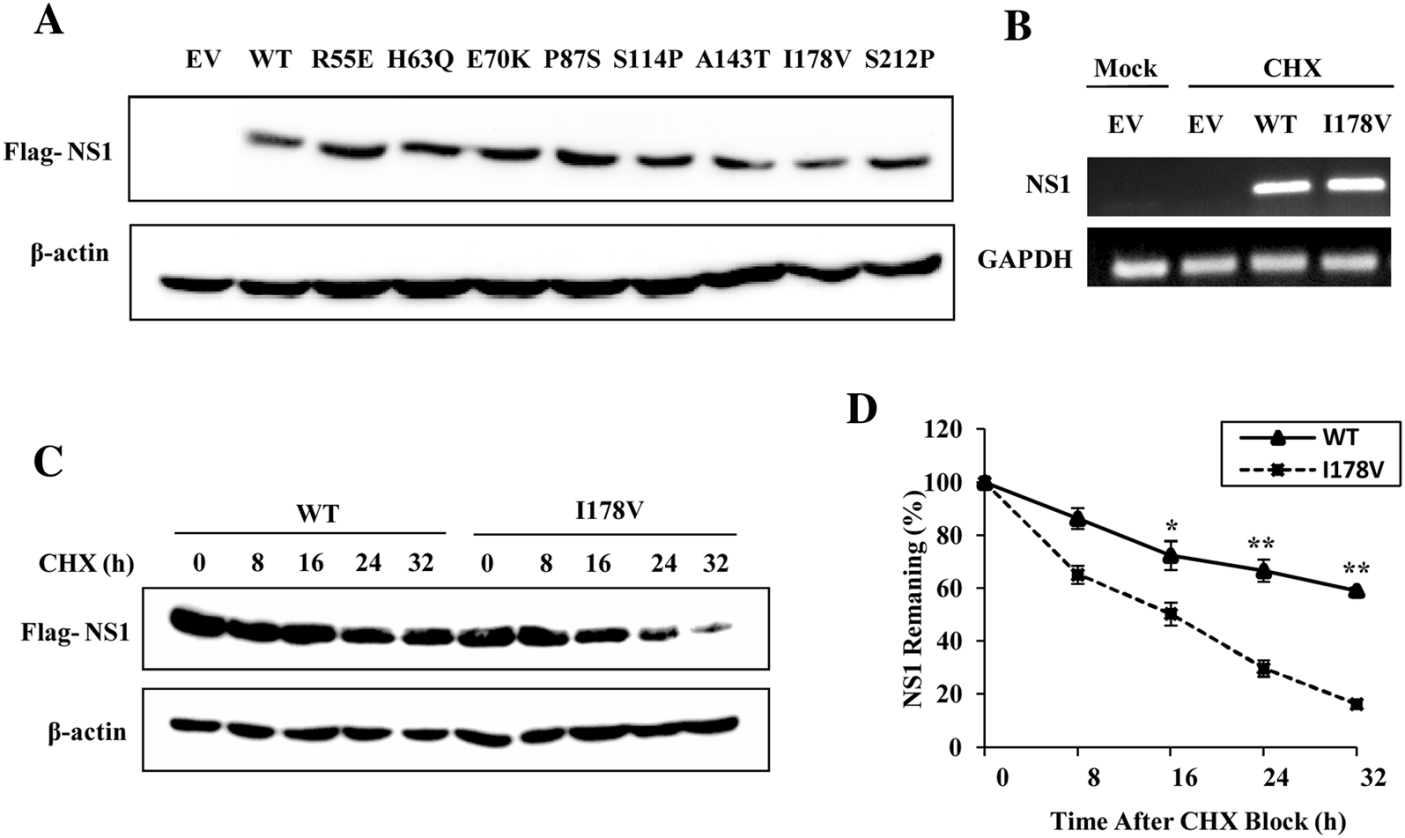
**I178V mutation decreases stability of H7N9 NS1 protein. A** 293T cells were transfected with plasmids expressing H7N9 NS1-WT (WT) or NS1 mutants (R55E, H63Q, E70K, P87S, S114P, A143T, I178V, S212P) for 24 h, followed by Western blotting to detect the NS1 protein levels. The results are representative of three independent experiments. **B** 293T cells were transfected with plasmids expressing H7N9 NS1-WT (WT) or NS1-I178V (I178V) as described in (**A**). Then the mRNA levels of NS1 were detected by RT-PCR. **C** Half-lives of NS1-WT (WT) and NS1-I178V (I178V) were examined. 293T cells were transfected with plasmids expressing H7N9 NS1-WT (WT) or NS1-I178V (I178V) as described in (**A**), then the cells were treated with CHX (100 μg/mL). At the indicated times after treatment, cells were harvested and cell extracts were prepared for Western blotting to analyze NS1 protein levels. **D** NS1 levels in (**C**) were quantitated by densitometry, and normalized to β-actin levels. Plotted are the average levels from three independent experiments. ^*^P < 0.05, ^**^P < 0.01.

After the eight H7N9 NS1 mutants were constructed, they were transfected into 293T cells to detect the protein expression levels. Unexpectedly, we found that protein expression of NS1 mutant I178V was less than that of the other seven mutants (Figure 5A). To determine whether the reduction of NS1-I178V protein level is the consequence of a decrease in its mRNA level, 293T cells transfected with NS1-WT or NS1-I178V were examined by RT-PCR. We observed that there were no statistically significant differences between NS1-WT and NS1-I178V mRNA levels (Figure 5B), indicating that the decrease in the amount of NS1-I178V protein may occur at the post-transcription, translation or protein degradation levels. Previous study has shown that amino acid substitutions of NS1 protein would affect its steady-state level [30], we next estimated the relative half-lives of NS1-WT and NS1-I178V protein to examine whether NS1-I178V protein underwent degradation in the cell. 293T cells transfected with plasmids expressing NS1-WT or NS1-I178V were treated with CHX for the indicated times, and Western blot analysis was performed to measure the amount of NS1 protein remaining at various time points, and to calculate the rates of NS1 remaining in the cells as percentages of NS1 protein levels at the zero time point. As shown in Figures 5C and D, the half-life of NS1-WT protein was more than 32 h, whereas I178V mutation dramatically shortened the half-life of NS1 to approximately 16 h, suggesting that I178V mutation promoted NS1 protein degradation in the cells.

### I178V mutation induces NS1 protein degradation through proteasome pathway

**Figure 6:**
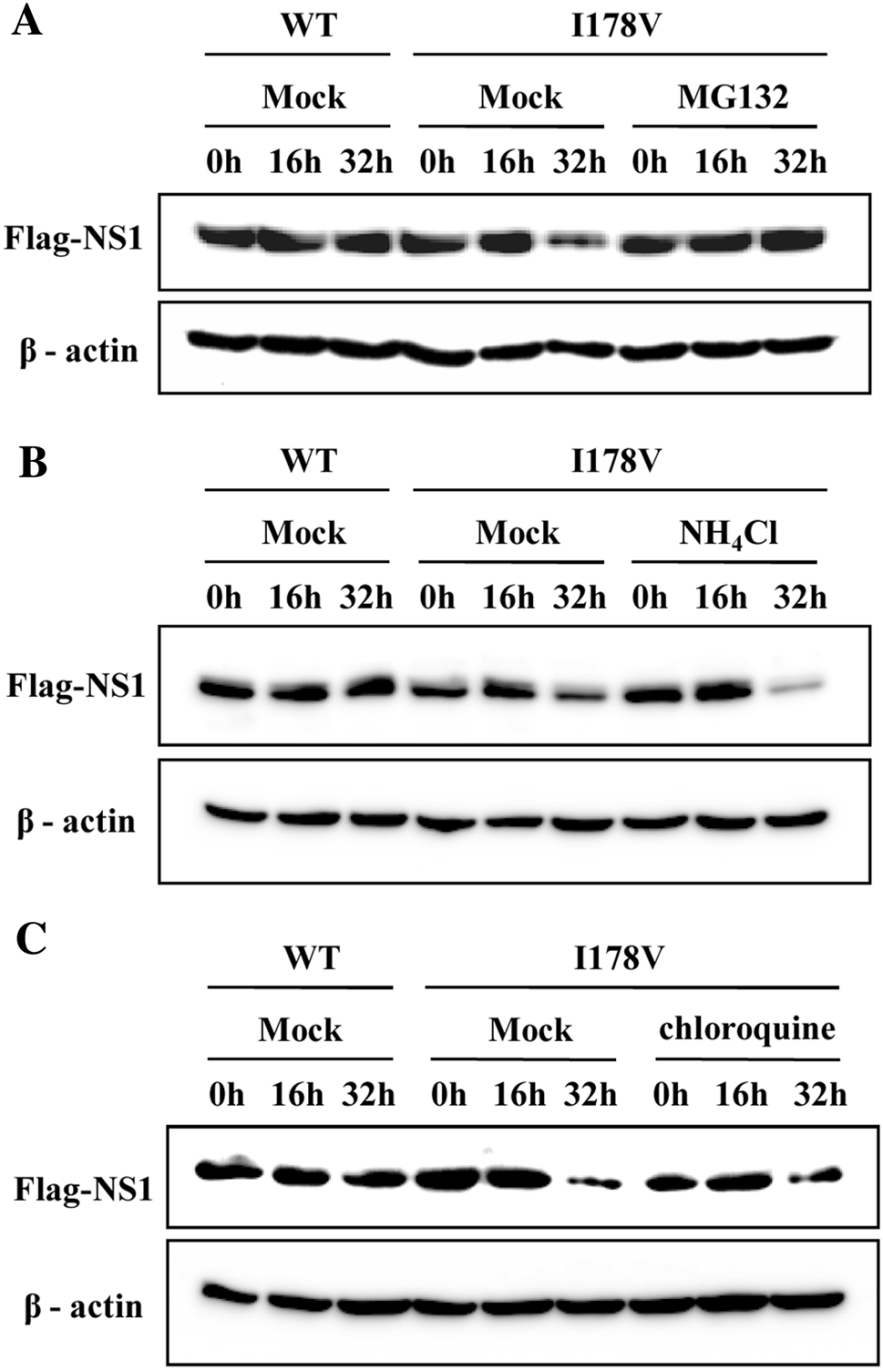
**I178V mutation induces proteasome degradation of NS1 protein. A**–**C** 293T cells transfected with plasmids expressing H7N9 NS1-WT (WT) or NS1-I178V (I178V) were treated with CHX (100 μg/mL), then were either mock treated or treated with MG132 (10 μM) (**A**), NH4Cl (20 mM) (**B**) or chloroquine (50 μM) (**C**). At the indicated times, cells were harvested and analyzed by Western blotting with the indicated antibodies. Shown are representative data of three independent experiments with similar results.

There are two major protein degradation systems, the proteasome pathway and the lysosome pathway [40]. To investigate by which pathway the degradation of NS1-I178V protein is regulated, cells transfected with plasmids expressing NS1-WT or NS1-I178V were cotreated with CHX and distinct proteasome inhibitor MG132 or lysosome inhibitors NH_4_Cl and chloroquine. We found that treatment with MG132 reversed the effect of I178V mutation on NS1 protein stability and clearly increased NS1-I178V protein levels (Figure 6A). In contrast, no significant changes for NS1-I178V protein levels were observed in cells treated with either NH_4_Cl or chloroquine (Figures 6B and C). These data suggested that I178V mutation-induced NS1 degradation is by the proteasome pathway and not via the lysosome pathway.

### P212S and V178I mutation in PR8 NS1 protein enhanced virulence and promoted virus replication in vivo

**Figure 7:**
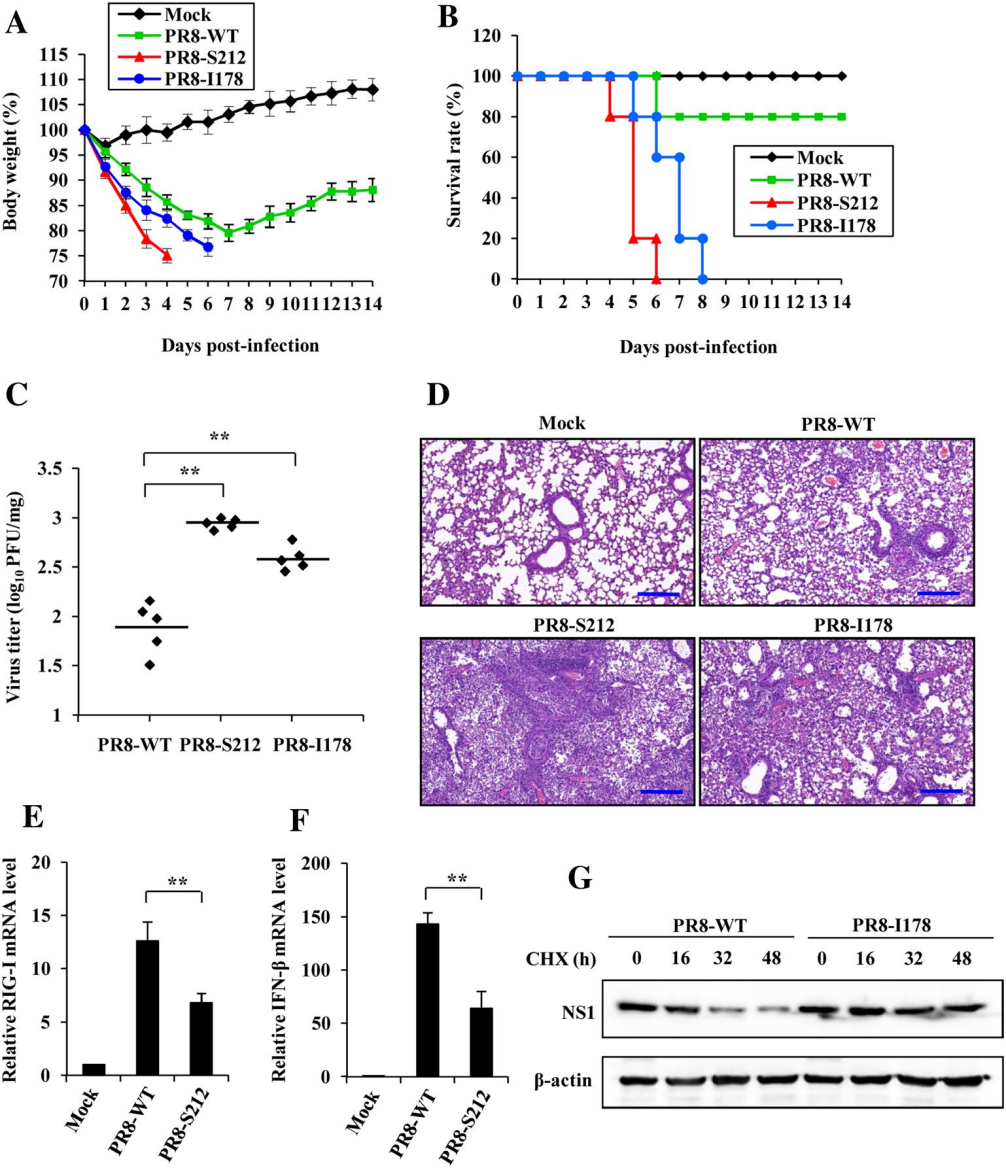
**P212S and V178I mutation in NS1 of PR8 virus enhances its virulence in vivo. A** Shown is the body weight change of mice mock infected or infected with PR8-WT, PR8-S212 or PR8-I178 (1 × 10^4^ PFU/mouse). Body weight was measured daily. The results are shown as mean percentage weight changes from three independent experiments. **B** Survival rate of mice infected with PR8-WT, PR8-S212 or PR8-I178 (*n* = 10 mice per group). Mice were monitored for up to 14 days. During this period, mice were sacrificed when they displayed severe unrelieved distress, hind limb paralysis or excessive weight loss (25% weight loss from initial body weight). **C** Shown is the viral load measured on day 5 post-infection in the lungs of mice infected with PR8-WT, PR8-S212 or PR8-I178. **D** Mice were mock infected or infected with PR8-WT, PR8-S212 or PR8-I178 for 5 days. Shown are representative micrographs of mouse lungs stained with hematoxylin and eosin (HE). Bars, 200 μm. **E**, **F** Three groups of mice (five mice per group) were mock infected or infected with PR8-WT and PR8-S212 for 5 days. Then the mRNA expression level of RIG-I (**E**) and IFN-β (**F**) was detected by quantitative real-time PCR. In each experiment, the value observed in mock-infected cells was normalized to 1. The error bars represent the s.e.m. **G** A549 cells were infected with PR8-WT and PR8-I178 for 16 h, then the cells were treated with CHX (100 μg/mL) for indicated times, followed by Western blotting to detect the NS1 protein levels. The results are representative of three independent experiments. **P < 0.01.

To further evaluate the effect of amino acids mutation at 212 and 178 sites of NS1 protein on virus growth and virulence in vivo, we generated mutated PR8 virus harboring P212S or V178I mutation in the NS1 protein using reverse genetics approach. Meanwhile, the mutations introduced in the NS1 did not result in amino acid changes in the viral NEP. Then mice were infected intranasally (i.n.) with PR8-WT virus or PR8 NS1 mutated virus (PR8-S212 or PR8-I178). Interestingly, we found that the mice infected with PR8-S212 or PR8-I178 displayed a faster body weight loss than those infected with PR8-WT during the viral infection (Figure 7A). Furthermore, the survival rate of infected mice was evaluated. We observed that all mice infected with PR8-S212 or PR8-I178 succumbed to viral infection by day 6 and day 8 respectively, whereas 80% of the mice infected with PR8-WT survived at the end of the experiments (at day 14 post-infection) under our experimental conditions (Figure 7B). Consistent with these observations, the plaque assay showed that the viral titers in lung tissues of mice infected with PR8-S212 or PR8-I178 were significantly higher than those infected with PR8-WT (Figure 7C), suggesting that NS1-P212S or NS1-V178I mutation generates a virus with an increased virulence in vivo.

In addition, the pathological changes of mouse lungs were examined following the virus infection. Notably, PR8-S212 or PR8-I178 infection led to a greater degree of lung injury reflected by macroscopic changes (Additional file 4). To further investigate the pathological features of the lungs, H&E staining was performed (Figure 7D). In the mouse lungs infected with PR8-WT, there were infiltration of inflammatory cells around the bronchiole and vessels, and a small number of inflammatory cells were seen in the alveoli. In contrast, abundant inflammatory cells were present in the alveoli and diffuse in the peribronchiolar and perivascular regions in the mouse lungs infected with PR8-S212 or PR8-I178. Moreover, the lumen of many bronchioles was congested and infiltrated with large amounts of lymphoid cells and severe alveolar collapse was observed after PR8-S212 infection. Finally, we examined the NS1 function and protein stability in mice and cells infected with PR8-S212 or PR8-I178 viruses. Consistently, we found that PR8-S212 infection significantly decreased RIG-I and IFN-β expression compared with that in PR8-WT infected mice. We demonstrated that the NS1 I178 protein was more stable than NS1 WT protein by using PR8-I178 virus and PR8-WT virus for in vitro studies (Figures 7E–G). These results further indicate that P212S and V178I mutation in the NS1 protein enhanced PR8 virulence, and caused more severe organ damage.

## Discussion

The novel H7N9 influenza virus which can transmit from poultry to humans continues to circulate in China, and causes mild to lethal human respiratory disease. Recently, H7N9 virus appeared to evolve to be highly pathogenic in birds in some cases [14–16], making it a growing concern that H7N9 influenza virus may cause lethal diseases both in humans and birds. Previous studies have shown that the H7N9 influenza virus is more virulent in human than the 2009 pandemic H1N1 influenza virus, and amino acid substitutions in H7N9 virus proteins may contribute to the enhanced virulence [6]. For example, G219S and K58I combined mutations or three-amino-acid mutations (V186G/K-K193T-G228S or V186N-N224K-G228S) in H7N9 HA protein resulted in high affinity to α-2,6-linked sialic acid (SA) and increased HA stability [10, 41], and a R292K mutation in H7N9 NA protein facilitated drug resistance through decreasing the binding interaction with oseltamivir, the most commonly used anti-influenza drug [11]. In addition, either E627K or V598T/I substitution in PB2 contributes to the higher virulence of H7N9 influenza virus than the 2009 pandemic H1N1 influenza virus and the seasonal H3N2 influenza virus through enhancing replication and pathogenicity of H7N9 influenza virus in mammals [8, 42, 43].

Influenza virus NS1 protein plays a critical role in blockade of the host innate immune response, and is considered as a major virulence factor. Stepwise changes in NS1 also contribute to the mammalian adaptation of avian influenza A viruses [44]. In this study, we constructed the plasmid expressing the novel H7N9 influenza A virus NS1 protein, and characterized it as an efficient IFN antagonist. Previous studies have shown that an amino acid substitution in the novel H7N9 influenza A virus NS1 protein could confer CPSF-binding ability, thus promoting virus replication and virulence [45]. Here, to identify new residues in H7N9 NS1 that are associated with virus virulence, we screened eight substitutions including R55E, H63Q, E70K, P87S, S114P, A143T, I178V and S212P that distinguish the NS1s of avian-origin influenza viruses from those of human strains. The subsequent function analysis showed that S212P mutation seriously impaired NS1 ability of inhibiting host interferon response, indicating that S212 residue in H7N9 NS1 protein may contribute to the higher virulence of H7N9 influenza virus.

Previous evidences have suggested that influenza virus NS1 protein can inhibit the induction of Type I IFN by decreasing RIG-I activation [18, 46]. In agreement with this, overexpression of H7N9 NS1 significantly decreased the expression of RIG-I mRNA induced by WSN or PR8 delNS1 virus infection. However, S212P mutation decreased the ability of NS1 to suppress RIG-I expression. Given the previous reports and the fact that influenza virus NS1 protein can inhibit host gene expression by binding and inhibiting the cellular factor CPSF30 [47], the verification whether the NS1 mutation S212P affects its binding to CPSF30 should be further studied. By blocking gene expression in infected cells, the H7N9 NS1 protein suppresses the expression of IFN-α, IFN-β, IL-28 and several ISGs, whereas this ability was destroyed due to S212P substitution. Consistent with the data, S212P mutation could no longer promote virus growth, and the viral titer was slightly lower in NS1-S212P transfected cells than that in NS1-WT transfected cells.

In order to survive better in the host cell, influenza A virus NS1 protein has been undergoing continuous evolution through mutation [48]. NS1 residue 212 is a proline in the vast majority of influenza A viruses, while instead a relative majority of human-isolated H7N9 viruses have a serine at position 212. Residue 212 is in the intrinsically disordered C-terminal tail of NS1 [49]. Beside the S212P substitution that decreased the ability of H7N9-NS1 to inhibit the host innate immunity, we also identified another NS1 amino acid substitution I178V, which can significantly decrease the steady state of NS1 protein. Previous study has shown that influenza virus NS1 could enhance its stability by sumoylation, and NS1 K221 is the major SUMO1 acceptor site, since NS1 K221R mutation displayed a significant decrease of sumoylation [30]. Here, we showed that I178 site in H7N9 NS1 protein was also implicated in its steady state. When the isoleucine at 178 site was changed to valine, which is a common amino acid in the NS1 of low pathogenic influenza virus, H7N9 NS1 protein became unstable and underwent degradation. In addition, we also identified that I178V mutation led to NS1 degradation through proteasome pathway. We postulated that I178V mutation might have altered the structure of the NS1 protein and caused its degradation. It is reasonable to assume that the I178 site increases H7N9 NS1 protein stability and is responsible for an enhanced impediment of host innate immunity.

To analyze the importance of the residues 212 and 178 in NS1 protein on virus growth and virulence, two recombinant PR8 viruses incorporating the mutation P212S (PR8-S212) or V178I (PR8-I178) were generated by reverse genetics. Interestingly, we observed that the mice infected with PR8-S212 or PR8-I178 exhibited faster weight loss, shorter survival time, increased viral load, and more severe organ damage as compared with those infected with PR8-WT, suggesting that P212S or V178I mutation in PR8 NS1 protein increased virulence, which probably correlates with increased antagonism to innate immunity and steady state of NS1 protein respectively.

Therefore, in this study, we identified two novel amino acid substitutions that have important effects on H7N9 NS1 function and virus replication, which reminds us to pay more attention to influenza virus surveillance and assessing mutations affecting the pathogenicity of circulating influenza viruses.

## Additional files

**Additional file 1.** **Amino acid sequence alignment between five influenza virus strains.** The NS1 amino acid sequence of influenza virus A/Anhui/1/2013 (H7N9) was aligned with that of four other influenza virus strains, A/Hong Kong/156/97 (H5N1), A/WSN/1933 (H1N1), A/Puerto Rico/8/1934 (H1N1) and A/California/04/2009 (H1N1) using MegAlign software of DNAStar (Lasergene version 7.1) package. The variable amino acids of NS1 protein between highly pathogenic (H7N9 and H5N1) and low pathogenic (WSN, PR8, CA04) influenza virus were marked with the red dashed box.

**Additional file 2.** **Effects of NS1 S212P mutation on the expression of innate immunity related genes.** (A, B) 293T cells (A) or A549 cells (B) transfected with plasmids expressing H7N9 NS1-WT (WT), NS1-S212P (S212P) or EV were infected with WSN virus (MOI = 1) for 12 h, followed by RT-PCR to detect the mRNA levels of indicated genes. (C, D) 293T cells transfected with plasmids expressing H7N9 NS1-WT (WT), NS1-S212P (S212P) or EV were infected with PR8 (C) or PR8 delNS1 (D) virus as described in (A), followed by RT-PCR to detect the mRNA levels of indicated genes. (E, F) 293T cells transfected with plasmids expressing H7N9 NS1-WT (WT), NS1-S212P (S212P) or EV were infected with WSN (E) or PR8 delNS1 (F) virus (MOI = 1) for 12 h. Then the mRNA levels of IL-6 and IL-1β were detected by RT-PCR.

**Additional file 3.** **Effects of NS1 S212P mutation on the expression of RIG-I, MDA5 and TLR3.** (A, B) 293T cells transfected with plasmids expressing H7N9 NS1-WT (WT), NS1-S212P (S212P) or EV were infected with WSN (A) or PR8 delNS1 (B) virus (MOI = 1) for 12 h, followed by RT-PCR to detect the mRNA levels of indicated genes.

**Additional file 4.** **Macroscopic changes in lungs of mice infected with PR8-WT, PR8-S212 or PR8-I178.** Mice were mock infected or infected intranasally with PR8-WT, PR8-S212 or PR8-I178 (1 × 10^4^ PFU/mouse) for 5 days. Then mice were sacrificed, and the lungs were collected. Shown are representative images from three independent experiments.
