# Supplementary figures and images for: Identification of two residues within the NS1 of H7N9 influenza A virus that critically affect the protein stability and function
Source: Vet Res. 2018 Oct 1;49:98. doi: 10.1186/s13567-018-0594-y (PMC6389221; doi:10.1186/s13567-018-0594-y)

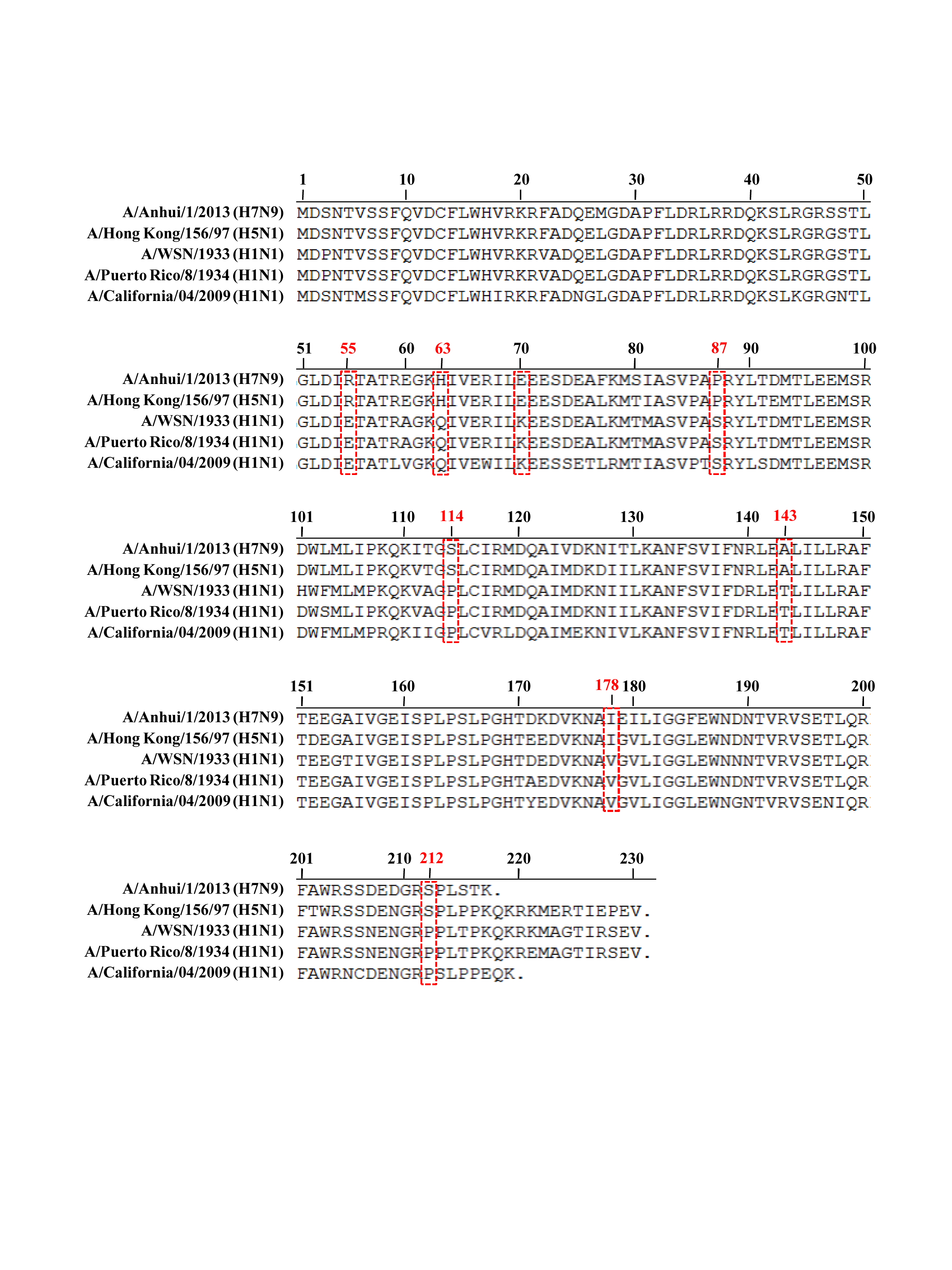

Supplement: Supplementary file 1 — Additional file 1. Amino acid sequence alignment between five influenza virus strains. The NS1 amino acid sequence of influenza virus A/Anhui/1/2013 (H7N9) was aligned with that of four other influenza virus strains, A/Hong Kong/156/97 (H5N1), A/WSN/1933 (H1N1), A/Puerto Rico/8/1934 (H1N1) and A/California/04/2009 (H1N1) using MegAlign software of DNAStar (Lasergene version 7.1) package. The variable amino acids of NS1 protein between highly pathogenic (H7N9 and H5N1) and low pathogenic (WSN, PR8, CA04) influenza virus were marked with the red dashed box. [file 13567_2018_594_MOESM1_ESM.tif]

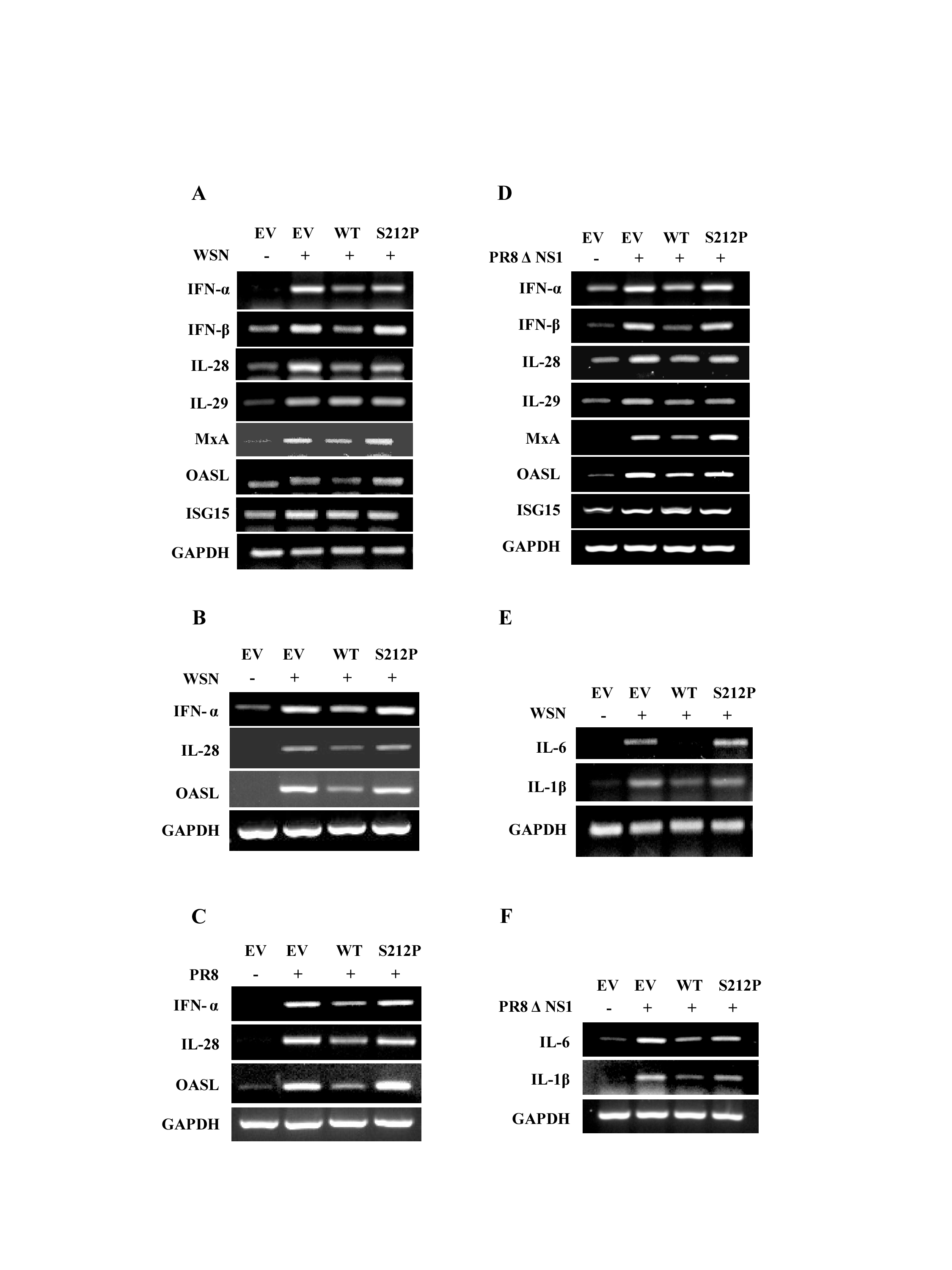

Supplement: Supplementary file 2 — Additional file 2. Effects of NS1 S212P mutation on the expression of innate immunity related genes. (A, B) 293T cells (A) or A549 cells (B) transfected with plasmids expressing H7N9 NS1-WT (WT), NS1-S212P (S212P) or EV were infected with WSN virus (MOI = 1) for 12 h, followed by RT-PCR to detect the mRNA levels of indicated genes. (C, D) 293T cells transfected with plasmids expressing H7N9 NS1-WT (WT), NS1-S212P (S212P) or EV were infected with PR8 (C) or PR8 delNS1 (D) virus as described in (A), followed by RT-PCR to detect the mRNA levels of indicated genes. (E, F) 293T cells transfected with plasmids expressing H7N9 NS1-WT (WT), NS1-S212P (S212P) or EV were infected with WSN (E) or PR8 delNS1 (F) virus (MOI = 1) for 12 h. Then the mRNA levels of IL-6 and IL-1β were detected by RT-PCR. [file 13567_2018_594_MOESM2_ESM.tif]

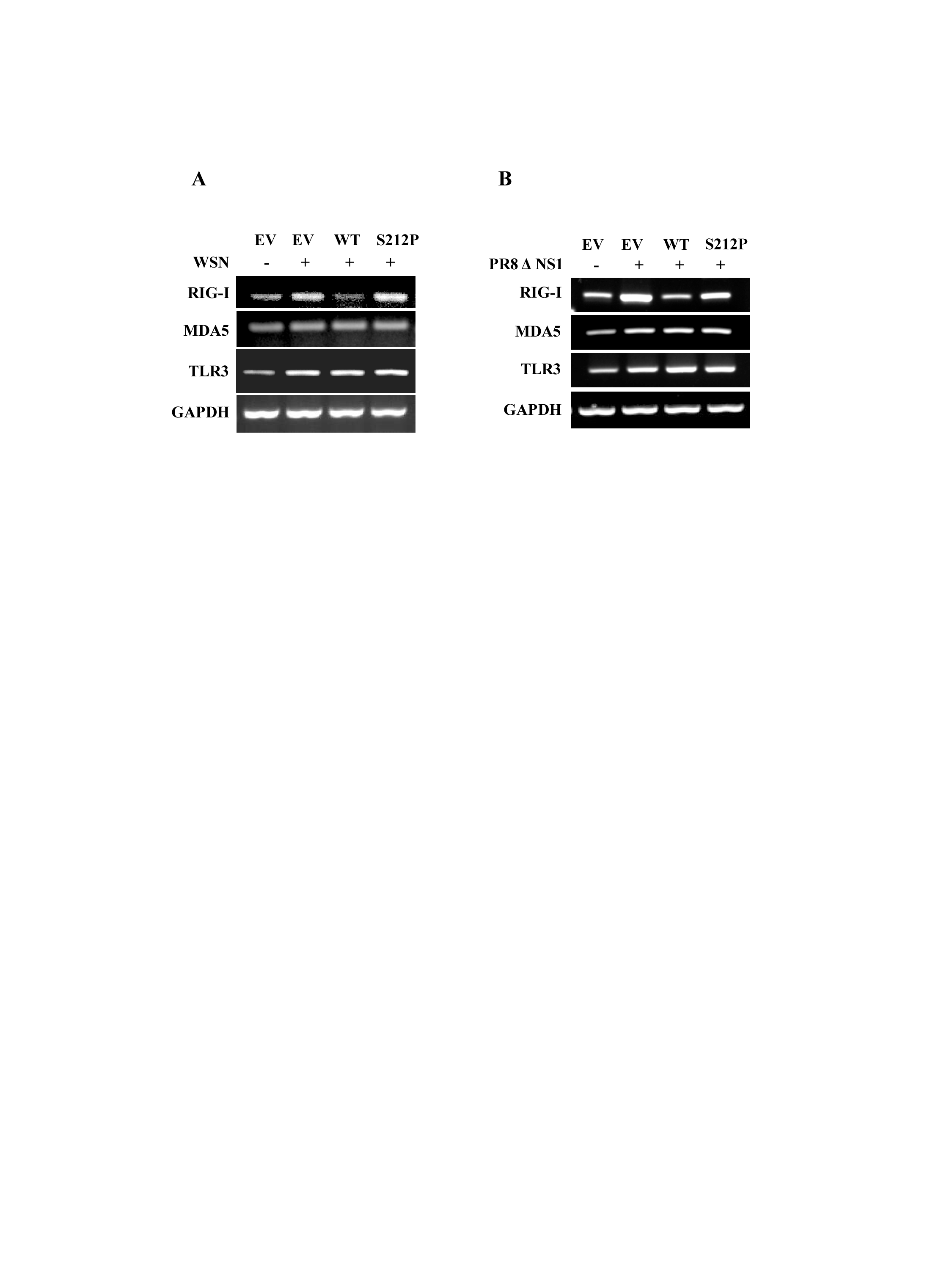

Supplement: Supplementary file 3 — Additional file 3. Effects of NS1 S212P mutation on the expression of RIG-I, MDA5 and TLR3. (A, B) 293T cells transfected with plasmids expressing H7N9 NS1-WT (WT), NS1-S212P (S212P) or EV were infected with WSN (A) or PR8 delNS1 (B) virus (MOI = 1) for 12 h, followed by RT-PCR to detect the mRNA levels of indicated genes. [file 13567_2018_594_MOESM3_ESM.tif]

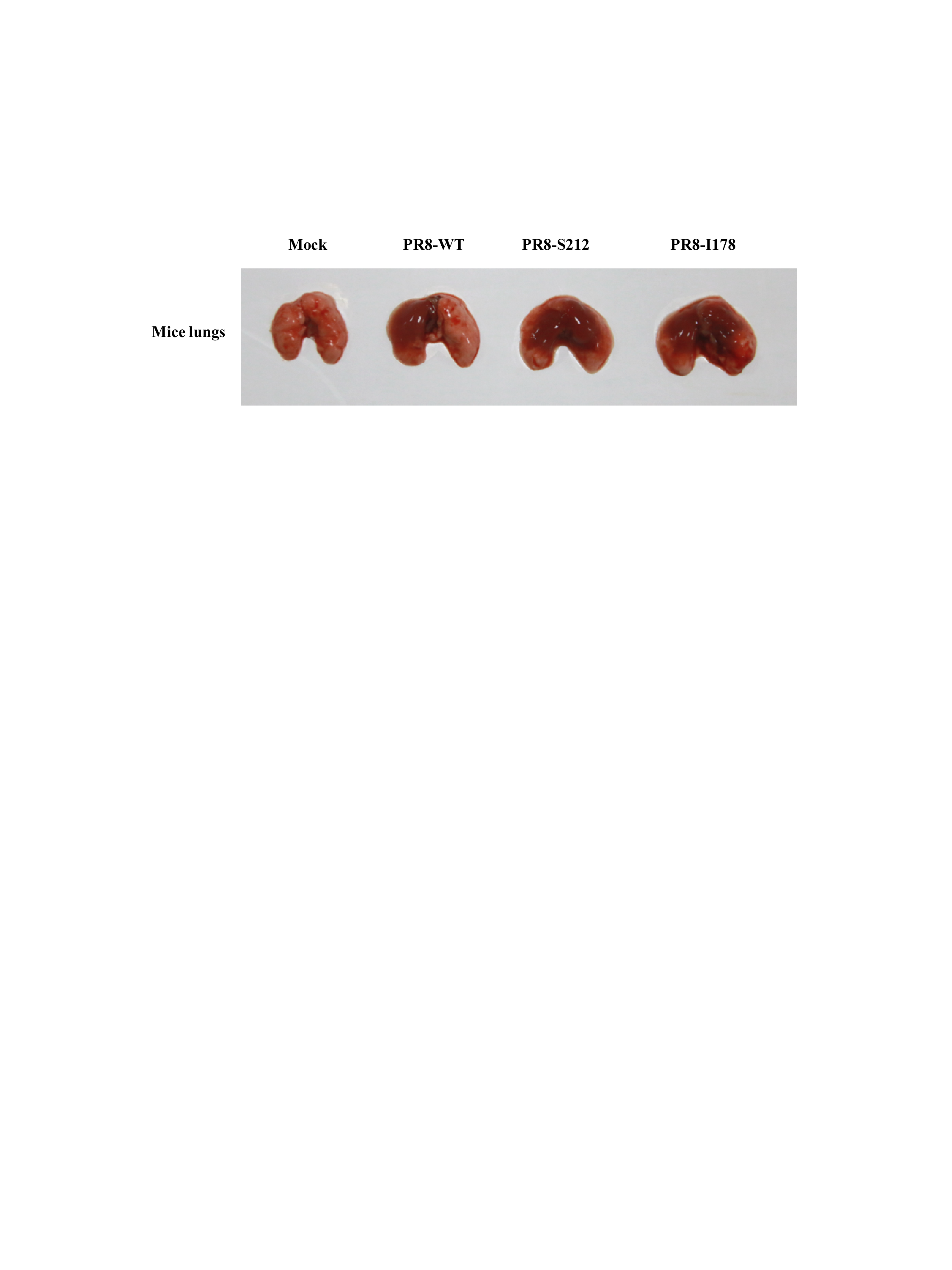

Supplement: Supplementary file 4 — Additional file 4. Macroscopic changes in lungs of mice infected with PR8-WT, PR8-S212 or PR8-I178. Mice were mock infected or infected intranasally with PR8-WT, PR8-S212 or PR8-I178 (1 × 10^4^ PFU/mouse) for 5 days. Then mice were sacrificed, and the lungs were collected. Shown are representative images from three independent experiments. [file 13567_2018_594_MOESM4_ESM.tif]
